# Emerging Roles of Extracelluar Vesicles Derived from Bacteria, Mammalian or Plant Cells in the Pathogenesis and Clinical Application of Neurodegenerative Diseases

**DOI:** 10.3390/biom14030312

**Published:** 2024-03-06

**Authors:** Yihong Li, Chenglong Zhou, Huina Liu, Ting Cai, Huadong Fan

**Affiliations:** 1 Ningbo No. 2 Hospital, Ningbo 315099, China; yihongli@ucas.ac.cn (Y.L.); liuhuina@ucas.ac.cn (H.L.); caiting@ucas.ac.cn (T.C.); 2 Ningbo Institute of Life and Health Industry, University of Chinese Academy of Sciences, Ningbo 315000, China; 3 Laboratory of Nanopharmacology Research for Neurodegeneration, Department of Research and Development of Science and Technology, Ningbo Institute of Life and Health Industry, University of Chinese Academy of Sciences, Ningbo 315000, China; 4 College of Biological & Environmental Sciences, Zhejiang Wanli University, Ningbo 315100, China; 2022881047@zwu.edu.cn; 5 Laboratory of Dementia and Neurorehabilitation Research, Department of Research and Development of Science and Technology, Ningbo Institute of Life and Health Industry, University of Chinese Academy of Sciences, Ningbo 315000, China

**Keywords:** extracellular vesicles, outer membrane vesicles (OMVs), plant-derived exosome-like nanoparticles (PDELNs), gut dysbiosis, gut–brain axis, microglia, neuroinflammation, neurodegeneration

## Abstract

A growing number of studies have indicated that extracellular vesicles (EVs), such as exosomes, are involved in the development of neurodegenerative diseases. Components of EVs with biological effects like proteins, nucleic acids, or other molecules can be delivered to recipient cells to mediate physio-/pathological processes. For instance, some aggregate-prone proteins, such as β-amyloid and α-synuclein, had been found to propagate through exosomes. Therefore, either an increase of detrimental molecules or a decrease of beneficial molecules enwrapped in EVs may fully or partly indicate disease progression. Numerous studies have demonstrated that dysbiosis of the gut microbiota and neurodegeneration are tightly correlated, well-known as the “gut–brain axis”. Accumulating evidence has revealed that the gut bacteria-derived EVs play a pivotal role in mediating microbe–host interactions and affect the function of the “gut–brain axis”, which subsequently contributes to the pathogenesis of neurodegenerative diseases. In this review, we first briefly discuss the role of EVs from mammalian cells and microbes in mediating the progression of neurodegenerative diseases, and then propose a novel strategy that employs EVs of plants (plant cell-derived exosome-like nanoparticles) for treating neurodegeneration.

## 1. Introduction

Neurodegenerative diseases are most commonly characterized by the aggregation of misfolded proteins due to improper post-translational modification of proteins like TDP-43 in amyotrophic lateral sclerosis/frontal temporal lobe dementia (ALS/FTLD), α-synuclein (α-syn) in Parkinson’s disease (PD), and β-amyloid (Aβ) in Alzheimer’s disease (AD) [1,2,3]. Nearly all types of cells, including prokaryotic and eukaryotic cells, secrete extracellular vesicles (EVs) with a diameter ranging approximately from 10 nm to 200 nm. As the mechanism of EV generation may vary among different species depending on parental cells, and because the exact process by which EVs are released from bacterial cells or plant cells remains unclear, here we only introduce an example of how exosomes are generated by mammalian cells. Generally, the exosomes are produced in a process involving the invagination of a double plasma membrane and the formation of intracellular multivesicular bodies (MVBs) containing intraluminal vesicles (ILVs) [4,5]. Finally, the MVBs undergo a process resembling exocytosis to release mature exosomes [6]. Evidence has shown that exosomes play a key role in propagating disease-associated proteins related to neurodegeneration within the brain [7,8,9]. Exosomes contain various components of their parental cells, including nucleic acids, lipids, and proteins from the cytoplasm and the surface membrane, as well as cellular metabolites, which can be taken up by their target cells. For instance, exosomes can either carry detrimental factors released from neurons to induce inflammation in glial cells, facilitating the progression of neurodegeneration [10], or engage in neuroprotective signaling transduction [11,12,13,14].

## 2. Extracellular Vesicles from Mammalian Cells

### 2.1. Behaviors and Functions of Mammalian EVs

The uptake of EVs is mediated in several ways (Table 1), including endocytosis, phagocytosis, and direct fusion with the plasma membrane. It has been demonstrated that the anchor proteins of the surface membrane of EVs can interact with membrane receptors on recipient cells, and this “ligand–receptor” interaction mediates the uptake of EVs by their target cells [15]. To address this mechanism, investigators used specific inhibitors or antibodies to block receptor–ligand interactions, revealing that the uptake of EVs was significantly hampered in a variety of cell types, which demonstrated that receptor-mediated endocytosis contributes to the uptake process of EVs [16,17,18,19,20]. Additionally, another study showed that some EV membranes were able to fuse directly with the plasma membrane of the recipient cells by labelling melanoma cell-derived exosomes with the lipid fluorescent probe Octadecyl Rhodamine B Chloride (R18) [21]. These studies together suggested that there are several known mechanisms underlying EV uptake, and the cells of different types or with different functions may choose a different manner of EV uptake to complete EV-mediated intercellular communication. Below is a table that lists several types of EV uptake.

### 2.2. Role of EVs of Mammalian Cells in Neurodegenerative Diseases

EVs play a double role in the central nervous system. On the one hand, disease-associated proteins can be propagated by EVs shuttled between different cells. As the disease develops, these proteins spread from one focal point in the brain to a larger scope of neuronal regions, accelerating the progression of neurodegeneration [22,23]. EVs containing disease-associated proteins involved in Prion disease, Parkinson’s disease (PD), Alzheimer’s disease (AD), and amyotrophic lateral sclerosis (ALS) have all been found in the cerebral spinal fluid (CSF) and blood of patients affected by these disorders [24]. Prion diseases are a group of rare progressive neurodegenerative diseases, including Creutzfeldt–Jakob disease (CJD), Gerstmann–Straussler–Scheinker disease, and kuru [25,26]. It is now widely accepted that the misfolding of the host-encoded prion protein, PrP^C^, into a disease-associated transmissible form, PrP^SC^, results in the transmission of pathology not only between cells but also from one region to another [27,28]. Both forms of prion proteins were found to be shuttled by exosomes [29]. Exosomal PrP^SC^ was found to transmit protein aggregation in rabbit kidney epithelial cells [30]. Subsequent in vivo experiments showed that exosomes derived from prion-infected mice were able to transmit aggregation to naïve mice [31,32]. For many years, PrP^SC^ involved in prion disease was the only known transmissible protein for the spread of disease, but recent studies using both animal and cellular models have confirmed that other proteins related to neurodegeneration are also transmissible. This includes α-synuclein in PD, and tau and Aβ in AD [33]. For example, EVs are an efficient carrier of α-synuclein aggregation and propagation between neurons, thus promoting the progression of PD [34]. Furthermore, EVs circulating in the blood and CSF of patients with PD have been found to be highly enriched with α-synuclein and are remarkably correlated with the stage of the disease [35]. For AD, it has been shown that neurotoxic, oligomeric forms of Aβ protein are wrapped in EVs isolated from brain tissue, and these vesicles can mediate the inter-neuronal propagation of Aβ [34]. To testify the critical role of EVs in AD development, an in vivo study revealed that injecting 5xFAD mice (AD model mice) with neutral sphingomyelinase 2 (nSMase2), an inhibitor of exosome secretion, significantly reduced amyloid plaque formation in the brain [31]. In addition, another study demonstrated that, as carriers of Aβ, astrocytes-derived extracellular vesicles (ADEVs) are involved in the pathogenesis of AD [36]. In the brain, astrocytes phagocytose too much fibril Aβ42 to digest them, which causes a severe accumulation of intracellular Aβ. To avoid further intracellular stress, astrocytes release undigested fibrils of Aβ42 via EVs, which would, in turn, lead to severe neurotoxicity in neighboring neurons [37]. Also, in ALS patients, astrocytes can generate EVs, which are toxic and lead to adjacent motor neuron death [38]. Furthermore, ADEVs mediate the propagation of neuroinflammation as well as regulate mutual signaling between the brain and the immune system. In a mouse model of inflammatory brain injury, ADEVs rapidly enter the peripheral circulation, inducing an acute peripheral cytokine response to accelerate the migration of peripheral leukocytes to the brain, thereby triggering neuroinflammation [39]. The above experimental data suggested that ADEVs in the peripheral blood might serve as a source of biomarkers for neurological disorders. As the EVs circulating in the blood are likely to be derived from various tissues throughout the body, the isolation of cell type-specific EVs can provide us with information about a certain pathological status. Namely, analyzing the contents of EVs derived from neurons or glial cells in the blood would help to identify novel biomarkers related to neurodegenerative diseases [40]. 

On the other hand, EVs act as a scavenger that can remove aggregation-prone misfolded proteins of cellular/intercellular space, exerting a neuroprotective effect [41]. As shown by investigators, the correctly folded prion protein (PrP^C^) on EVs could trap neurotoxic β-amyloid (Aβ) to promote its fibrillation. In this case, the role of PrP^C^-contained exosomes is to remove Aβ to diminish its neurotoxicity and prevent the accumulation of misfolded proteins [31]. Additionally, in order to take advantage of the neuroprotective role of mammalian cell-derived EVs, numerous studies have concentrated on the therapeutic effect of stem cell-derived EVs, especially on mesenchymal stromal cell-derived EVs (MSC-EVs) [42,43,44,45,46,47]. It was initially found that mesenchymal stromal cells (MSCs), isolated from bone marrow or adipose tissues, can significantly mitigate neurodegeneration [46,48]; later, investigators confirmed that even MSC-EVs themselves can strongly alleviate cognitive impairment caused by brain injury, stroke, or neurodegeneration [14,49,50], accompanied by obvious neuron regeneration throughout the ventricular region, cingulated gyrus, and hippocampus [51,52,53]. MSCs have the strong ability to migrate and differentiate, interacting with brain parenchyma to release vascular endothelial growth factors (VEGFs), nerve growth factors (NGFs), brain-derived neurotrophic factor (BDNFs), and other bioactive molecules to promote the regeneration of blood vessels and nerves, and the reconstruction of neural synapses, as well as to prevent neuron apoptosis [54,55,56,57]. In addition, MSCs can restrict the release of inflammatory molecules like prostaglandins and interleukins to minimize neuroinflammation [58,59]. The above beneficial effects that MSCs display depend on their paracrine function rather than on direct interaction with the diseased site [44,49]. It was later verified that the conditioned medium of cultured MSCs showed a similar therapeutic effect to that of MSCs themselves [60,61]. More interestingly, EVs isolated from an MSCs-cultured medium showed almost the same protective effect as MSCs [59,62].

The exact mechanism underlying the neuroprotective role of MSC-EVs remains ambiguous. Generally, MSC-EVs have bioactive contents that include cytokines, growth factors, signaling lipids, and regulatory microRNAs, which can influence tissue rehabilitation after injury, infection, or disease [59]. For example, over 900 varieties of protein molecules in MSC-EVs have been identified using proteomics technology, including neprilysin, a protease that can degrade Aβ oligomer [63]. In addition, Egor A. and colleagues found that MSC-EVs exert a neuroprotective role via preventing calcium overload in an PI3K/AKT-dependent manner [14]. 

### 2.3. The Potential of MSC-EVs as a Biogenic Drug for Treating AD

In the pathogenesis of AD, a high level of homocysteine in plasma (hyperhomocysteinemia, HHcy) is an independent risk factor [64,65,66,67]; HHcy AD mice show an increased Aβ level in the brain [68]. In homocysteine metabolism, insufficiency of 5-methlytetrahydrofolate (the active form of folate) would result in an accumulation of its upstream substrate, homocysteine [69], which is consistent with another study showing that a folate-deficient diet can also accelerate brain amyloidosis in an AD mouse model [70]. Meanwhile, investigators have indicated that high folate intake decreases the risk of AD [71]. However, sufficient dietary intake of folate does not mean that it is efficiently delivered to the brain; in particular, the blood–brain barrier (BBB) excludes most of the free folate in the plasma. The efficient delivery of folate to the brain parenchyma largely depends on the specific recognition of folate-receptor α (FRα), which is shuttled by EVs derived from choroid plexus epithelial cells [72,73,74]. Therefore, only with the help of FRα shuttled by exosomes can folate can be smoothly transported through the BBB to reach the neurons or glia. Since previous studies have shown that MSCs contain a high level of FRα, it is a strong possibility that FRα might appear in MSC-EVs. In fact, it was independently demonstrated by our lab that there is a high abundance of FRα in MSC-EVs (unpublished data). 

**Figure 1:**
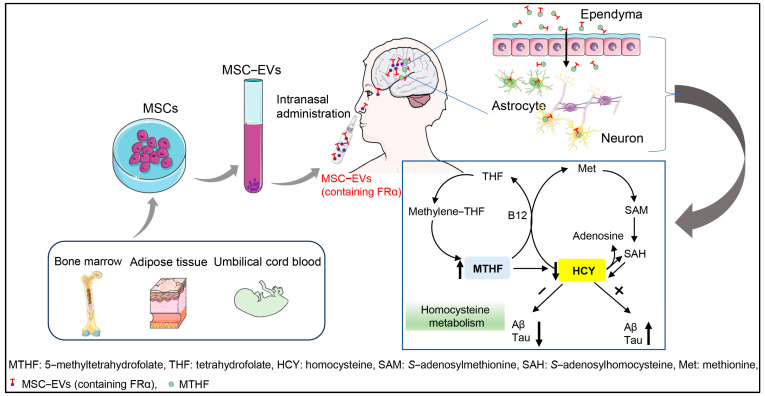
A possible mechanism underlying the neuroprotective role of MSC-EVs in Alzheimer’s disease. Abbreviations: MSCs, mesenchymal stromal cells; MSC-EVs, mesenchymal stromal cell-derived extracellular vesicles; MTHF: 5-methyltetrahydrofolate; THF, tetrahydrofolate; HCY, homocysteine; SAM, *S*-adenosylmethionine; SAH, *S*-adenosylhomocysteine; Met, methionine.

In summary, based on the above evidence, as shown in Figure 1, we hypothesize that MSC-EVs might be used for AD treatment through supplementing with FRα (MSC-EVs containing FRα), thus facilitating folate uptake by the brain parenchyma and finally blocking HHcy-facilitated amyloidosis in the brain. In AD patients, one can obtain MSCs from bone marrow, adipose tissue, or umbilical cord blood, and then isolate EVs from MSCs-cultured medium. Upon intranasal administration, the MSC-EVs could easily penetrate the BBB and release folate into the brain parenchyma. The key mechanism of this process is to modulate homocysteine metabolism by affording efficient folate through EVs-mediated folate transportation. In fact, a clinical trial (NCT04388982) on the intranasal administration of MSC-EVs to AD patients is already being conducted by another research group [75].

## 3. Bacterial EVs

Humans are colonized by multiple commensal organisms. Our gastrointestinal tract provides a residence for both beneficial and pathogenic microorganisms, both of which release bilayer lipid membrane nanovesicles (outer membrane vesicles, OMVs) of spherical morphology with a diameter ranging from 20 to 400 nm [76]. Being different from mammalian EVs, OMVs contain bacteria-specific lipopolysaccharides and peptidoglycans except for regular DNA, RNA, and protein [77,78].

### 3.1. Behaviors and Functions of Bacterial EVs

The behaviors of OMVs and their biological effects are complicated. Imbalance in the composition of the beneficial and pathogenic bacteria, known as dysbiosis, is considered as a major contributor to inflammatory bowel disease. Also, emerging evidence has indicated that OMVs play a key role in the development of inflammatory diseases. OMVs usually carry immunogenic molecules, including lipopolysaccharides, peptidoglycan or related proteins, that can be recognized by specific receptors expressed in the host cells, which eventually either exacerbate pathological conditions or promote host colonization and confer protective immunity (Table 2) [79]. Generally, there are two methods by which OMVs enter into the host cell as follows: the first can be seen in *pseudomonas aeruginosa*-secreted OMVs, the plasma of which directly fuses with its host cell’s membrane and then releases the carried contents; the second is endocytosis, seen in *Escherichia coli*, through which the contents of OMVs directly enter into the host cytoplasm [80]. For the subsequent biological effects, OMVs from pathogenic bacteria can exacerbate infection by either suppressing the immune response or over-exacerbating it. For example, OMVs from *Pseudomonas aeruginosa* carry a variety of virulent factors, including peptidoglycan hydrolase, phospholipase C, alkaline phosphatase, protease, and hemolysin [81]. These OMV-enwrapped detrimental factors, in combination with Lipopolysaccharide (LPS), elicit an inflammatory response with a bacterial strain-specificity [82]. Furthermore, researchers also found that only OMVs from isolated from *Escherichia coli* cultures can trigger an inflammatory response [83]. In addition to the directly detrimental effect of OMVs, they can also elicit the pathological function by interacting with the other microorganism. For instance, human *Bacteroides fragilis* can protect the host from colitis by releasing a single microbial molecule polysaccharide (PSA); however, OMVs secreted from *Bacillus subtilis* may disrupt such protection by degrading polysaccharides [84].

### 3.2. Role of Bacterial EVs in Neurodegenerative Diseases

Substantial evidence has revealed a strong connection between the gut and the brain—referred to as the gut–brain axis—and the composition of the gut microbiota and their derivative OMVs have an important impact on neurological disorders [88,89]. It is reported that, by using mice overexpressing α-synuclein, more α-synuclein aggregates were deposited in the brains of control mice compared to those of germ-free mice, and oral administration of specific bacterial metabolites to germ-free mice enhanced neuroinflammation and motor symptoms, suggesting that the gut microbiota and their secretions in the form of OMVs transmission might be an important contributor to α-synuclein pathology and microglia activation in Parkinson’s disease [90]. Similarly, there appeared to be significant changes of the composition of the gut microbiota in AD model (5xFAD) mice compared to that of control mice at the age of 6 months, which were characterized by a dynamic increase in the abundance of pro-inflammatory molecule-generating bacteria such as *Aspergillus*, *Mimicryptosporium*, and *Terratula* [91]. Consistent with the viewpoint that dysbiosis of gut microbiota might be a risk factor for neurodegeneration, an excellent study conducted by Teng et al. elucidated that isoamylamine (IAA), a metabolite secreted by gut bacteria, promotes age-related cognitive degeneration by inducing microglia death [92]. A reasonable mechanism by which IAA is transported to the brain might be the high permeability of the intestinal mucosal barrier due to exposure to detrimental factors (including OMV content or pro-inflammatory molecules) resulting from the alterations of gut microbiota. Usually, increased permeability of the gut mucosal barrier is accompanied by susceptibility to colitis. Studies have demonstrated that OMVs released by beneficial *Bifidobacterium fragilis* deliver PSA to the dendrite cells (DCs) of the intestine to mediate mutual interactions between the bacteria and the gut immune system, leading to the inhibition of pro-inflammatory cytokine production and the prevention of colitis [87]. In addition, OMVs from *Lactobacillus rhamnosus GG* can increase the expression of antimicrobial peptides and tight junction proteins of the intestine to prevent gut barrier destruction [93]. Based on the current findings detailed above, the health of the gut may strongly influence the functions of the brain, while the metabolites, particularly the OMVs of microorganism in the gut, probably dominate the pathogenesis of neurodegeneration. 

## 4. Plant Derived Exosome-Like Nanoparticles

Plant-derived exosome-like nanoparticles (PDELNs) are nanosized vesicles usually isolated from edible fruits or vegetables [94], which have huge potential for clinical applications compared to exosomes from mammalian cells or bacteria. The highlighted feature of PDELNs includes non/low toxicity, ideal biodistribution, efficient bioavailability, and high yield [95,96,97]. Most PDELNs are structurally similar to mammalian exosomes, with an apparent spherical structure of lipid bilayers. In comparison with cholesterol, glycosphingolipids, ceramides, and phosphatidylserine are composed of a mammalian exosome lipid bilayer [98,99,100,101], and the membranes of PDELNs are enriched with phosphatidic acid (PA), phosphatidylcholines (PC) digalactosyldiacylglycerol (DGDG), and monogalactosyldiacylglycerol (MGDG), providing inherent mammalian-cell-regulating activities [102,103].

### 4.1. Biological Functions of PDELNs

It has been reported that plant exosomes can be absorbed by intestinal microorganisms, exemplified by the composition of microorganisms in the feces of C57BL/6 mice that were altered by oral administration with grape exosome-like nanoparticles (GELNs) [103]. GELNs can also regulate the growth of intestinal stem cells, as well as induce IL-22 expression through activation of the AHR pathway, thereby protecting the integrity of the intestinal barrier for the treatment of colitis [103,104]. Furthermore, Li and colleagues found that GELNs can easily pass through the blood–brain barrier in zebrafish and exert a protective effect on neurodevelopment [105]. In addition, grapefruit-derived nanoparticles (GDNs), which are selectively absorbed by intestinal macrophages, can ameliorate dextran sulfate sodium (DSS)-induced colitis by up-regulating heme oxygenase-1 (HO-1) expression and inhibiting the production of inflammatory factors [106]. Sulforaphane (SFN), enwrapped in broccoli-derived nanoparticles (BDNs), protects mice from colitis by inducing the production of anti-inflammatory factors through the AMPK pathway [107]. It has also been shown that, in addition to altering the composition of the intestinal microbiota, lemon exosome-like nanoparticles (LELNs) enhance the pharmaceutical effects of probiotics to inhibit *Clostridioides difficile* infection via AhR-dependent and AhR-independent pathways [108]. In addition to the critical role in regulating the intestinal microbiota, other studies found that the exosome-like nanoparticles from ginger (GELNs) can also inhibit *Porphyromonas gingivalis*—a periodontal pathogen that causes periodontitis—through their interaction with GELN cargo molecules including phosphatidic acid and miRNAs [109]. These current studies suggest that PDELNs may exert their biological function by restoring the gut barrier integrity (colitis), maintaining the normal composition of gut microorganisms, or through direct contact to inhibit the pathogenic bacteria. 

### 4.2. Potential Applications of PDELNs for Treating Neurodegenerative Diseases

Even though there are few studies showing the direct effect of PDELNs on the treatment of neurodegenerative disease, it is now certain that, as a novel therapeutic method, PDELNs have been demonstrated to be highly effective in treating inflammatory diseases like colitis [107], encephalitis [110], periodontitis [109] and so forth, among which the pathogenesis of colitis was considered to be strongly related to the dysfunction of the gut barrier and the dysbiosis of the gut microbiota. The above evidence raises the question of whether PDELNs could be considered a potential drug for the treatment of neurodegenerative disease.

The gut–brain axis is required for transducing detrimental signals from the gut to the brain. For instance, inflammatory factors resulting from leaky gut (with colitis) penetrate the blood–brain barrier (BBB), disrupting BBB integrity. As a result, the activated gut immune cells may be translocated to the brain to amplify neuroinflammation. In addition, inflammatory factors can also be transmitted through the vagal nerve that connects the gut and the brain, leading to increased neuroinflammation [111]. Regarding such a tight relation between gut and brain, any kinds of PDELNs that are effective for treating inflammatory gut diseases might also be useful for treating neurodegenerative diseases with the hallmark of apparent neuroinflammation. In particular, the efficacy of sulforaphane (SFN) had been systematically studied in neurodegenerative diseases, with an emphasis on its anti-inflammatory role [112]. Since SFN is enriched in broccoli-derived nanoparticles (BDNs) [107], it is highly possible that BDNs would also be effective in treating neurodegenerative diseases.

It is widely accepted that microglia play a pivotal role in the progression of Alzheimer’s disease (AD) [113,114]. Usually, normal proliferation, chemotaxis, and phagocytosis are required to remove excessive Aβ deposition [115]. However, while overactivated, microglia would release inflammatory cytokines to induce neuronal death [116,117]. In the process of microglia development, short-chain fatty acids (SCFA), one of the metabolites of intestinal microorganism, promote microglia maturation as well as their morphological and functional stabilization [118]. Interestingly, Teng and colleagues found that isoamylamine (IAA), a metabolite from pathogenic bacteria in the gut, promotes age-related cognitive dysfunction by inducing microglial cell death [92], which means that PDELNs might be effective for treating the cognitive decline associated with neurodegenerative diseases through restoring the function of microglia. 

Taken together, the possible mechanism of a therapeutic role of PDELNs in the treatment of neurodegenerative diseases is through rebalancing the composition of the gut microorganism, preventing the peripheral inflammatory factors entering the brain, thereby diminishing neuroinflammation. 

## 5. Conclusions and Future Perspectives

Although multiple factors (including genetic, environmental, dietary, and metabolic) contribute to the pathogenesis of neurodegenerative diseases such as Alzheimer’s disease (AD) and Parkinson’s disease (PD), and there exist significant heterogenicities among different individuals, two common factors have been agreed to date as follows: (1) the pathological hallmark is the presence of misfolded protein aggregations/depositions in the brain that are usually defined as proteinopathies; (2) progressive and long lasting neuronal cell death, as well as glia cell reactivation and neuroinflammation. Because emerging evidence has revealed a critical role of tissue-specific exosomes (mammalian EVs) in metabolizing disease-associated proteins, and because bacteria-derived exosomes (OMVs) can modulate gut microbiota thereby influencing gut inflammation and gut barrier integrity, one can postulate that these two varieties of EVs would strongly affect the progression of neurodegeneration. 

In the brain, both neurons and microglia generate EVs that carry excessive pro-fibrils of Aβ. Then, the pro-fibrils of Aβ are propagated to the extracellular space accompanied by the release of other payloads of EVs, resulting in Aβ deposition (amyloid plaque) and neuronal cell death. In the gut, dysbiosis of the microbiota leads to an imbalance of beneficial OMVs and pathogenic OMVs, disrupting the integrity of the gut barrier. The leaky gut facilitates more pro-inflammatory molecules and related peripheral immune cells to enter circulation, activating microglia and causing neuroinflammation. As a promising therapeutic approach, plant-derived exosome-like nanoparticles (PDELNs) may protect the brain through re-balancing the composition of the gut microbiota.

**Figure 2:**
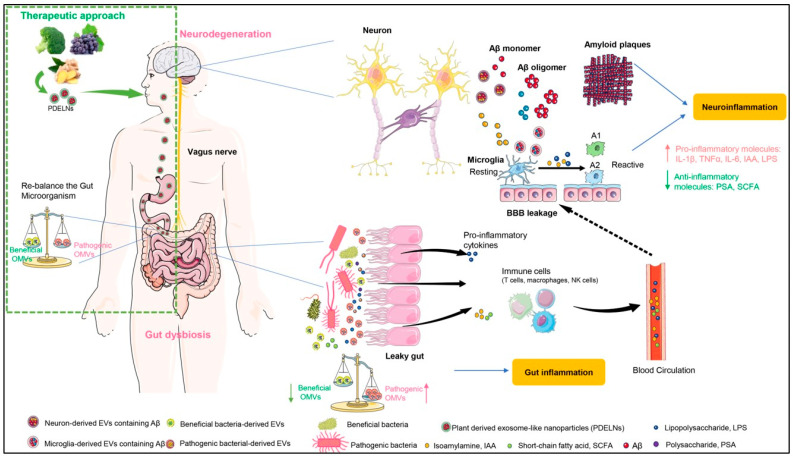
The role of extracellular vesicles from bacteria and their host in the pathogenesis of neurodegeneration, and a possible mechanism of plant-derived exosome-like nanoparticles as a novel approach to treating neurodegeneration. Abbreviations: OMVs, outer membrane vesicles; PDELNs, plant derived exosome-like nanoparticles; BBB, blood brain-barrier; IAA, isoamylamine; LPS, lipopolysaccharide; PSA, polysaccharide; SCFA, short-chain fatty acid.

One of the possible etiologies involving EVs is illustrated in Figure 2, which concludes that the imbalance of beneficial and pathogenic OMVs enhances the neuroinflammation of neurodegenerative disorders mediated by the gut–brain axis, suggesting a key role of not only mammalian cell-derived EVs but also bacteria-derived EVs in the development of neurodegeneration. However, the latest, more appealing studies conducted by several laboratories have revealed that PDELNs show promising therapeutic effects in terms of re-balancing the composition of the gut microbiota and relieving the inflammation of the gut and brain. This cross-kingdom EV communication suggests that one may use PDELNs as a potential therapeutic for treating neurodegenerative diseases. 

Although existing studies have provided a solid foundation on the mutual relations between OMVs and mammalian EVs, and between PDELNs and OMVs, respectively, the exact mechanism by which PDELNs reshape the gut microbiota, and whether PDELNs can directly protect the brain through communicating with neuron or glia-derived EVs remain ambiguous. In addition, further studies are needed to establish more effective contents of PDELNs to accelerate their clinical application to treating neurodegenerative disorders. 

## Figures and Tables

**Table 1:** Types of EV uptake.

Types of EVs Uptake	Examples	References
Endocytosis	1: CME. Recipient cells treated with chlorpromazine reduce the uptake of EVs, and chlorpromazine prevents the formation of lattice protein-coated pits in the plasma membrane.	[8,16]
	2: Phagocytosis. EVs were labelled with a fluorescent dye, and dendritic cells were found to have red fluorescence, confirming that they could phagocytose EVs.	[17]
	3: CDE. Endocytosis of EVs by CDE requires activation by dynamin2, which can be blocked by a specific inhibitor, leading to a significant reduction of internalization for EVs.	[18,19]
Membrane Protein interactions	1: Tetraspanins. Treatment of recipient cells with antibodies against tetraspanin reduces EV uptake by dendritic cells.	[20]
	2: Immunoglobulins. Naive T cells have been shown to internalize EVs mediated by the T cell receptor (TCR), CD28, and LFA-1. 3: Proteoglycan. Acetyl-heparin sulfate proteoglycan (HSPG) acts as a receptor for cancer cell-derived exosomes.	[6,21]
Cell surface membrane fusion	Purified exosomes from melanoma cells labeled with fluorescent lipid dye showed that some EV membranes were able to fuse directly with the plasma membrane of the recipient cells.	[15]

**Table 2:** Varieties of OMV secreted by pathogenic and beneficial bacteria.

Bacteria	Functions of OMVs	References
ETEC	ETEC OMVs can deliver ClyA, a pore-forming cytotoxin expressed by *E. coli* and some other enterobacteria.	[85]
*Pseudomonas aeruginosa*	OMVs from *Pseudomonas aeruginosa* contain multiple virulence factors, resulting a significant increase in the levels of inflammatory factors, triggering inflammation.	[81,82]
*H. pylori*	*H. pylori*-derived OMVs exert immunomodulatory effects by inducing the production of pro-inflammatory cytokines and promoting apoptosis of gastric epithelial and immune cells. They also induce apoptosis in human umbilical vein endothelial cells, which may promote atherosclerotic plaque formation.	[85]
*V. cholerae*	Cholera toxin (CT) is the main virulence factor of *Vibrio cholerae*, and OMVs may be the important carrier for transporting CT to epithelial cells.	[86]
*Bacteroides fragilis*	*Bacteroides fragilis* releases PSA by OMVs, inducing immunomodulatory effects, and prevents experimental colitis.	[87]
